# Charging failure due to pulse generator rotation after deep brain stimulation: a case report

**DOI:** 10.1093/omcr/omag137

**Published:** 2026-07-27

**Authors:** Di Lu, Mingming Zhao, Feng Yin

**Affiliations:** Department of Neurosurgery, Aerospace Center Hospital, No. 15 Yuquan Road, Haidian District, Beijing 100049, China; Department of Neurosurgery, Peking University Aerospace School of Clinical Medicine, No. 15 Yuquan Road, Haidian District, Beijing 100049, China; Department of Neurosurgery, Aerospace Center Hospital, No. 15 Yuquan Road, Haidian District, Beijing 100049, China; Department of Neurosurgery, Peking University Aerospace School of Clinical Medicine, No. 15 Yuquan Road, Haidian District, Beijing 100049, China; Department of Neurosurgery, Aerospace Center Hospital, No. 15 Yuquan Road, Haidian District, Beijing 100049, China; Department of Neurosurgery, Peking University Aerospace School of Clinical Medicine, No. 15 Yuquan Road, Haidian District, Beijing 100049, China

**Keywords:** deep brain stimulation, rechargeable implanted pulse generator, hardware complication, device rotation, Parkinson’s disease

## Abstract

A 54-year-old man with Parkinson’s disease presented with gait instability and difficulty charging his rechargeable deep brain stimulation (DBS) pulse generator. Chest radiography revealed horizontal rotation and anterior-to-posterior inversion of the device within the subcutaneous pocket, which was not seen in prior imaging. Surgical revision confirmed the device malorientation, and repositioning restored charging efficiency. This case highlights that pulse generator rotation, a rare but relevant hardware complication, can cause charging difficulties despite normal device interrogation. It underscores the importance of considering mechanical causes and using chest imaging in postoperative assessments.

## Introduction

Deep brain stimulation (DBS) is an established and effective therapeutic modality for Parkinson’s disease and other movement disorders, particularly in patients with motor fluctuations or symptoms refractory to pharmacological treatment. With continuing advances in neuromodulation technology, rechargeable implanted pulse generators (r-IPGs) have been increasingly adopted in DBS systems. Compared with non-rechargeable devices, r-IPGs offer several advantages, including prolonged battery longevity, a reduced frequency of replacement procedures, lower long-term healthcare costs, and a decreased risk of infection associated with repeated surgical interventions [1].

Despite these advantages, the widespread use of r-IPGs has introduced novel hardware-related complications [2]. Among these, postoperative charging difficulty has been increasingly recognised, although it remains infrequently reported in the literature. Charging difficulties may present as reduced charging efficiency, prolonged charging duration, or failure to establish effective coupling between the external charger and the implanted device, despite normal findings on device interrogation, including impedance measurements and lead integrity. Previous reports have suggested that such complications may be associated with device malorientation, excessive mobility within the subcutaneous pocket, patient manipulation, or increased soft-tissue thickness between the external charger and the r-IPG.

We report a case of Parkinson’s disease in which postoperative charging difficulty was attributable to horizontal rotation of the chest-implanted r-IPG. The charging dysfunction was successfully resolved by surgical revision with repositioning and secure fascial anchoring of the device. This case highlights the importance of recognising mechanical causes of r-IPG charging failure and provides practical insights into its clinical management.

## Case report

A 54-year-old man with a 10-year history of Parkinson’s disease was admitted because of progressive gait instability and increasing difficulty in charging his implanted DBS system (Table 1).

**Table 1:** Timeline of key clinical events.

Time	Event
2016	Diagnosis of Parkinson’s disease; commenced pharmacological treatment
March 2022	DBS implantation; r-IPG placed in right subcutaneous chest pocket; normal motor control and charging postoperatively
2025	Progressive gait instability; increasing charging difficulty with prolonged charging duration
Current admission (January 2026)	Normal device interrogation; chest radiography confirmed anterior-to-posterior inversion of r-IPG Surgical revision: r-IPG repositioned and anchored; normal orientation confirmed on postoperative imaging; charging restored immediately
3-month follow-up	Normal charging; device interrogation normal; no recurrence of hardware-related symptoms

He had been diagnosed with Parkinson’s disease in 2016 following the onset of progressive bradykinesia and rigidity, which initially responded well to levodopa therapy. Over time, he developed motor fluctuations despite optimisation of pharmacological treatment. In March 2022, he underwent DBS implantation (Pulse generator: PINS G102R), resulting in significant improvement in motor symptoms.

During the year preceding the present admission, he experienced gradual deterioration in balance. More notably, he reported increasing difficulty in charging the r-IPG, characterised by prolonged charging duration despite normal findings on device interrogation. He denied any recent trauma or conscious manipulation of the implanted device.

On neurological examination, he demonstrated hypophonic speech, reduced facial expression and bradykinesia during the medication ‘off’ state, with improvement in the ‘on’ state. No focal neurological deficits were identified.

**Figure 1:**
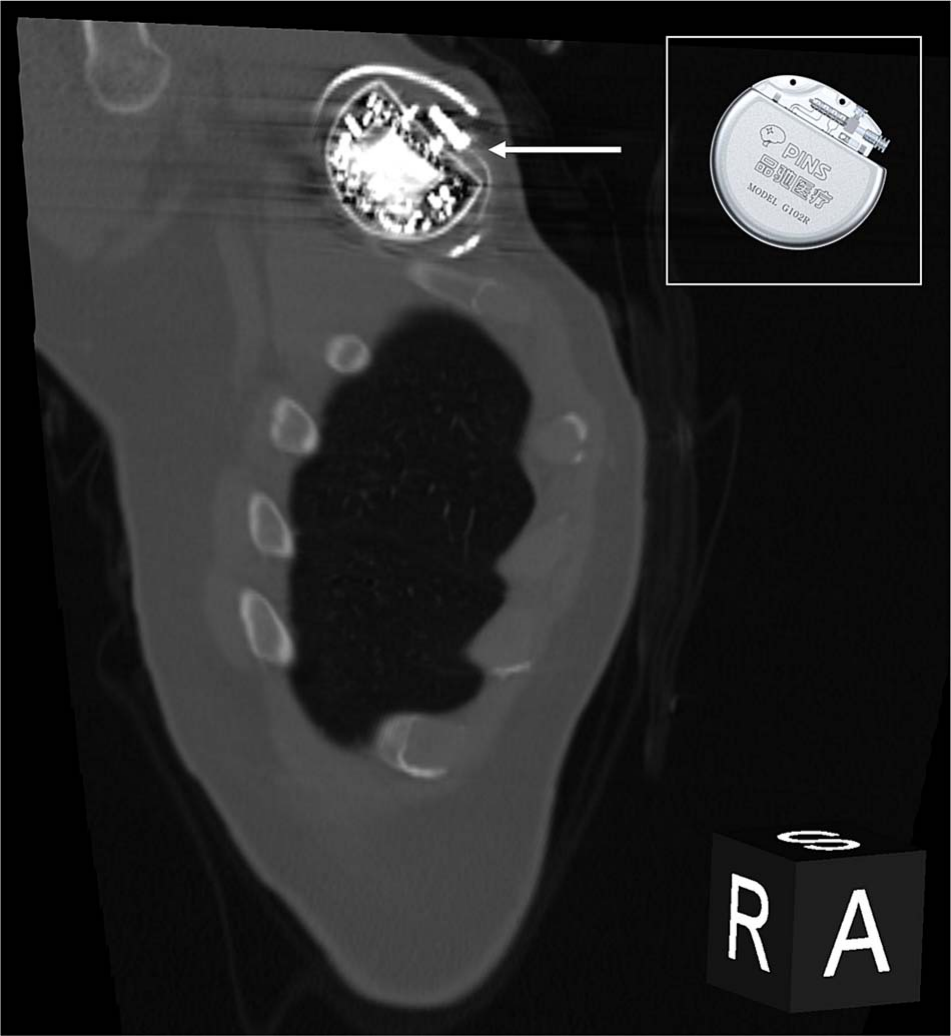
Chest CT performed after the initial deep brain stimulation implantation in 2022 demonstrating appropriate positioning of the rechargeable pulse generator (r-IPG) within the right subcutaneous chest pocket. The anterior, coil-facing surface of the device is oriented towards the overlying skin (upper right inset: Actual anterior view of the generator), representing the normal configuration required for efficient transcutaneous charging. The leads exit the device from the left side (white solid arrow) without coiling or displacement. No rotation or migration is identified.

Chest computed tomography (CT) performed shortly after the initial DBS implantation in 2022 demonstrated appropriate positioning of the r-IPG within the right subcutaneous chest pocket, with the device lying flat and its anterior, coil-facing surface oriented towards the overlying skin (Fig. 1). The leads exited the device from the left side without coiling or displacement, and the distance between the device and the skin surface was within normal limits.

**Figure 2:**
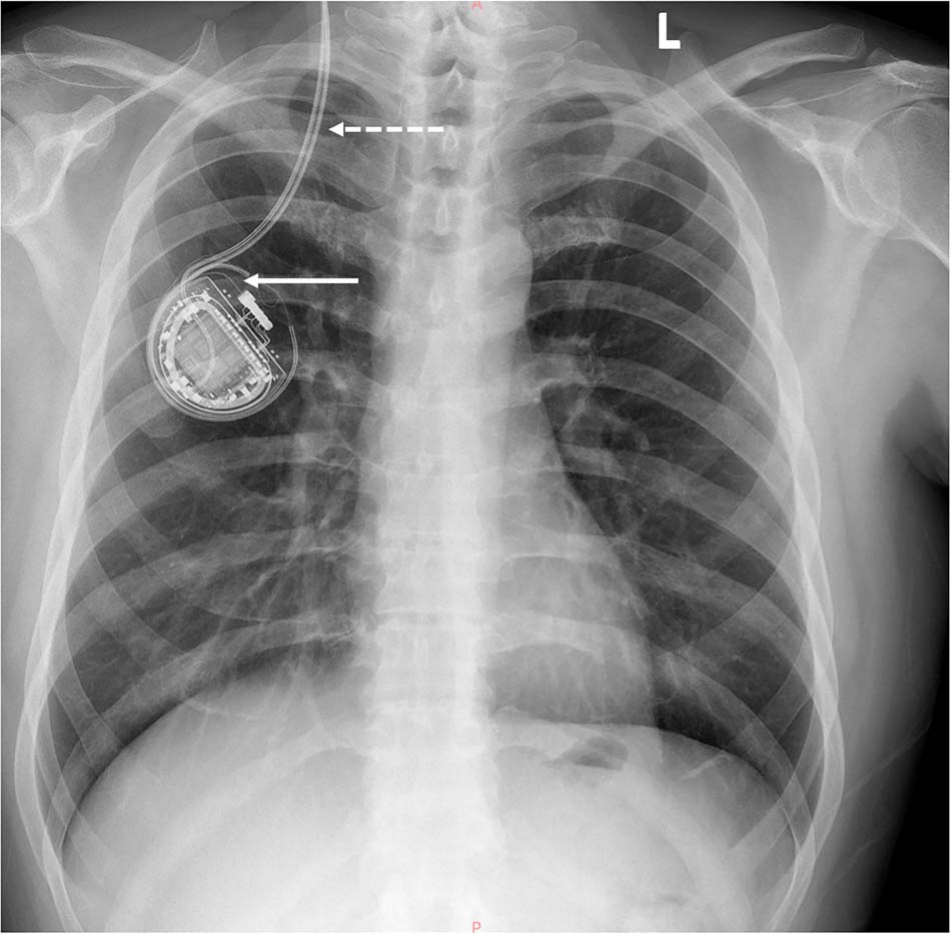
Chest radiograph obtained at the current admission demonstrating horizontal rotation of the r-IPG within the subcutaneous right chest pocket. The device has undergone approximately 180° of horizontal rotation, with the coil-receiving surface directed posteriorly away from the skin rather than anteriorly towards it, placing the charging coil in direct opposition to the skin and disrupting effective transcutaneous energy transfer. The leads exit the device from the right side (white solid arrow), with no evidence of lead coiling or displacement (white dashed arrow).

However, chest radiography obtained at the current admission revealed that the r-IPG had undergone horizontal rotation within the axial plane (Fig. 2). The device had undergone approximately 180° of horizontal rotation, such that its coil-receiving surface, which normally faces anteriorly towards the overlying skin, was now directed posteriorly away from the skin surface. This complete anterior-to-posterior inversion placed the charging coil in direct opposition to the skin rather than in the correct orientation for transcutaneous energy transfer, substantially increasing the effective distance between the external charger and the internal coil and thereby disrupting charging. The leads exit the device from the right side. No change in lead configuration or coiling was identified, indicating isolated device rotation without lead involvement.

**Figure 3:**
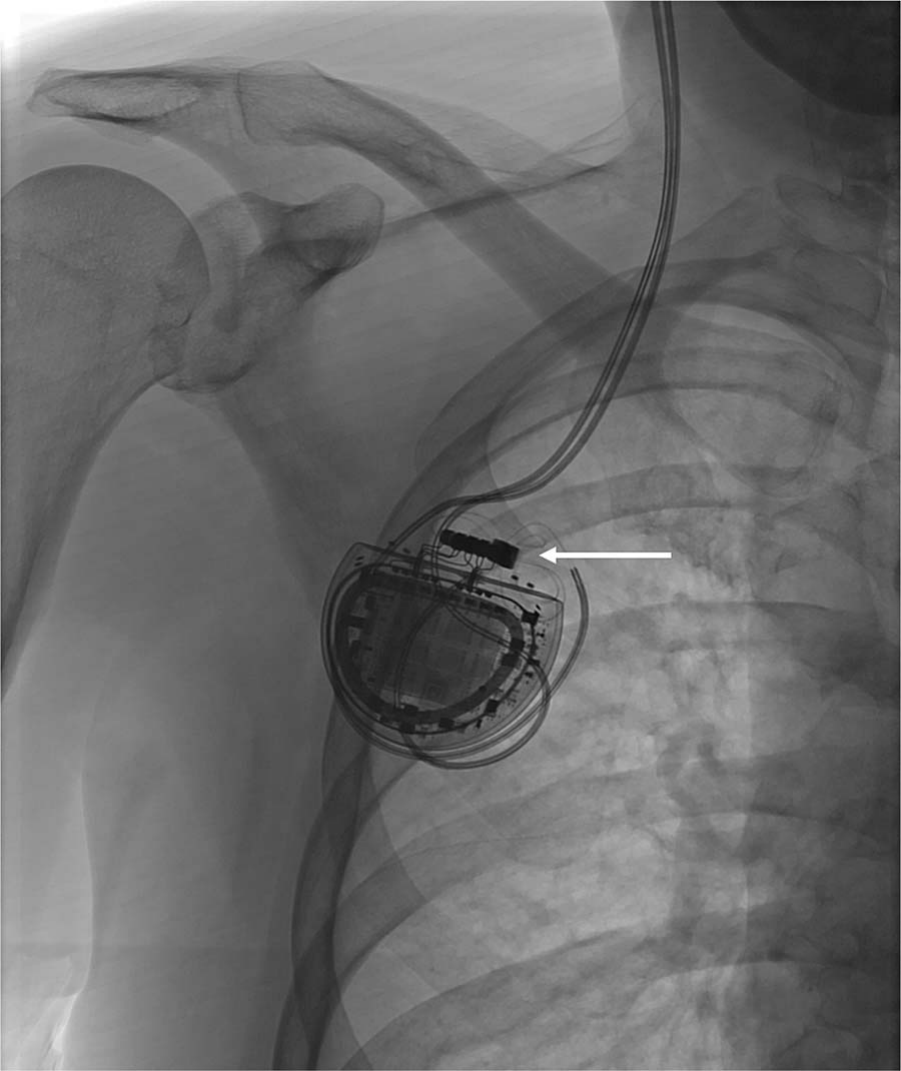
Postoperative chest radiograph following surgical revision demonstrating restoration of normal r-IPG orientation after repositioning and secure fascial anchoring. The coil-receiving surface has been returned to an anterior orientation and the leads exit the device from the left side (white solid arrow). No lead displacement or hardware abnormality is identified.

The patient subsequently underwent surgical revision. Intraoperative findings confirmed malorientation of the r-IPG within the subcutaneous pocket, consistent with the radiological findings. The device was repositioned to restore anterior orientation of the coil-receiving surface and securely anchored onto the underlying fascia to prevent recurrence. Postoperative imaging confirmed restoration of normal device orientation (Fig. 3). Charging efficiency improved immediately following the procedure, with complete resolution of the previously prolonged charging duration. At outpatient follow-up three months after revision surgery, the patient reported stable and reliable charging without recurrence, and device interrogation confirmed normal impedance and programme settings.

## Discussion

The r-IPGs have substantially improved the long-term management of patients undergoing DBS. However, their reliance on transcutaneous energy transfer renders correct device orientation and stable fixation essential for optimal function. Postoperative charging difficulty represents an uncommon but clinically significant hardware-related complication of DBS. As described in previous reports, patients typically present with prolonged charging time, reduced charging efficiency and normal impedance under device interrogation, suggesting a mechanical rather than electrical origin.

Malorientation or rotation of the r-IPG within the subcutaneous pocket is one of the principal mechanisms underlying this complication. In the case series reported by Li *et al*., charging difficulties occurred in approximately 0.4% of patients with r-IPGs, and most cases were associated with incorrect device orientation identifiable on chest radiography [3]. Surgical revision with repositioning and secure fascial anchoring reliably resolved the problem, further supporting the mechanical basis of the dysfunction.

This mechanism conceptually overlaps with Twiddler’s syndrome, a recognised hardware complication first described in cardiac implantable electronic devices and subsequently reported in DBS systems [4]. Twiddler’s syndrome is characterised by rotation of the implanted pulse generator within its pocket, often secondary to conscious or unconscious manipulation, resulting in lead displacement, device malfunction or hardware failure [5]. Reported risk factors include advanced age, female sex, obesity, excessive pocket size and inadequate fixation [6]. In contrast to classical Twiddler’s syndrome, which frequently involves lead coiling or fracture, our patient exhibited isolated horizontal rotation of the r-IPG without lead twisting or structural damage. Furthermore, whereas Twiddler’s syndrome has most commonly been described in elderly female patients, the present case involved a middle-aged male, thereby broadening the clinical spectrum of patients potentially at risk of device rotation. The absence of lead involvement and the atypical patient demographics distinguish the present case from most previously reported instances of both Twiddler’s syndrome and r-IPG charging failure, underscoring that this complication is not confined to the classical high-risk profile and should be considered across a broader patient population.

When a patient with a DBS r-IPG presents with charging difficulty, a structured diagnostic approach is warranted. Electrical causes, including lead fracture, connector malfunction, or impending battery depletion, can generally be excluded through formal device interrogation, which evaluates impedance, battery voltage, and programme integrity. In contrast, mechanical causes—most notably device malorientation or rotation within the subcutaneous pocket—may be entirely absent on device interrogation, as the electronic circuitry remains functionally intact despite the physical displacement. This distinction is clinically important: a normal interrogation result does not exclude a hardware complication and should not preclude further investigation when charging difficulty persists. Plain chest radiography is a rapid, accessible first-line investigation that can directly visualise device orientation and identify rotation, excessive mobility, or migration. Cross-sectional imaging with CT may provide additional detail regarding the depth and dimensions of the subcutaneous pocket, the spatial relationship between the device and the overlying skin, and the integrity of the lead system. The present case exemplifies this diagnostic pathway: normal device interrogation in the context of persistent charging failure prompted radiological evaluation, which identified the mechanical cause and guided definitive surgical management.

An additional important implication of this case concerns postoperative imaging strategy. In routine DBS follow-up, imaging often focuses primarily on cranial CT to confirm accurate intracranial electrode placement. However, this case illustrates that evaluation should not be limited to the cranial component of the system. Postoperative and follow-up chest imaging, particularly plain radiography, may be valuable in confirming correct orientation and stable positioning of the r-IPG. Early identification of device malorientation may prevent prolonged charging difficulty and avoid unnecessary device reprogramming or troubleshooting.

In the present case, chest radiography clearly demonstrated horizontal rotation of the r-IPG, and surgical revision with repositioning and secure fascial anchoring resulted in immediate restoration of charging efficiency. This outcome is consistent with previous observations that correction of device orientation is typically curative when a mechanical cause is identified. In conclusion, although rare, charging failure secondary to rotation of the implanted pulse generator represents a reversible hardware-related complication of DBS surgery. Recognition of this entity, awareness of its conceptual overlap with Twiddler-like mechanisms, and incorporation of chest imaging into postoperative assessment may facilitate early diagnosis and effective management.
